# Myostatin/AKT/FOXO Signaling Is Altered in Human Non-Ischemic Dilated Cardiomyopathy

**DOI:** 10.3390/life12091418

**Published:** 2022-09-12

**Authors:** Lea Hildebrandt, Maja-Theresa Dieterlen, Kristin Klaeske, Josephina Haunschild, Diyar Saeed, Sandra Eifert, Michael A. Borger, Khalil Jawad

**Affiliations:** Department of Cardiac Surgery, Heart Center, HELIOS Clinic, University Hospital Leipzig, 04289 Leipzig, Germany

**Keywords:** heart failure, cardiomyopathy, protein breakdown, E3 ligases, FOXO, MAFbx, MuRF1

## Abstract

**Simple Summary:**

Cardiac muscle-specific E3 ligases are enzymes that transfer ubiquitin to proteins to label them for protein degradation through the ubiquitin proteasome system. The E3 ligases MAFbx and MuRF1 are regulated by the myostatin/AKT/FOXO pathway that involves the molecules myostatin, AKT and the transcription factors FOXO1 and FOXO3. It is known that the expression of the two E3 ligases MAFbx and MuRF1 could be changed in cardiac diseases. The aim of this study was to clarify whether the myostatin/AKT/FOXO pathway as well as the expression of MAFbx and MuRF1 are changed in dilated cardiomyopathy of ischemic origin (IDCM) and dilated cardiomyopathy of non-ischemic origin (NIDCM) compared to a control group. MAFbx and transcription factor FOXO1 mRNA and protein expression as well as AKT mRNA and myostatin protein expression were decreased in patients with NIDCM compared to the control group. Apart from decreases of AKT and MAFbx mRNA expression, no significant differences were detected in patients with IDCM compared to the control group. Our results demonstrate that the myostatin/AKT/FOXO pathway is altered in patients with NIDCM, while patients with IDCM did not show substantial changes. The transcription factor FOXO1 seems to be an important drug target for regulating the expression of MAFbx in patients with NIDCM.

**Abstract:**

Disturbances in the ubiquitin proteasome system, and especially changes of the E3 ligases, are subjects of interest when searching for causes and therapies for cardiomyopathies. The aim of this study was to clarify whether the myostatin/AKT/forkhead box O (FOXO) pathway, which regulates the expression of the E3 ligases muscle atrophy F-box gene (MAFbx) and muscle ring-finger protein-1 (MuRF1), is changed in dilated cardiomyopathy of ischemic origin (IDCM) and dilated cardiomyopathy of non-ischemic origin (NIDCM). The mRNA and protein expression of myostatin, AKT, FOXO1, FOXO3, MAFbx and MuRF1 were quantified by real-time polymerase chain reaction and ELISA, respectively, in myocardial tissue from 26 IDCM and 23 NIDCM patients. Septal tissue from 17 patients undergoing Morrow resection served as a control. MAFbx and FOXO1 mRNA and protein expression (all *p* < 0.05), AKT mRNA (*p* < 0.01) and myostatin protein expression (*p* = 0.02) were decreased in NIDCM patients compared to the control group. Apart from decreases of AKT and MAFbx mRNA expression (both *p* < 0.01), no significant differences were detected in IDCM patients compared to the control group. Our results demonstrate that the myostatin/AKT/FOXO pathway is altered in NIDCM but not in IDCM patients. FOXO1 seems to be an important drug target for regulating the expression of MAFbx in NIDCM patients.

## 1. Introduction

Cardiac remodeling as it occurs in cardiomyopathy is associated with molecular changes. The ubiquitin proteasome system is involved in the cardiac remodeling process because its major function is the degradation of proteins [1]. E3 ligases are part of the ubiquitin proteasome system and its ubiquitination machinery. Hundreds of E3 ligases are identified in humans, but only a few of them, including muscle atrophy F-box gene (MAFbx, also known as Atrogin-1) and muscle ring-finger protein-1 (MuRF1) are recognized to play a pivotal role in cardiomyocytes [2]. Klaeske et al. recently reported the differential downregulation of mRNA expression of the cardiac-specific E3 ligases MAFbx and MuRF1 after myocardial infarction (MI) and for MuRF1 mRNA after chronic heart failure (CHF) in a rodent model [3]. They hypothesized that the downregulation of mRNA expression of several E3 ligases after MI and in CHF is based on the changes in the myostatin/AKT/forkhead box O (FOXO) signaling pathway, suggesting a role of this pathway in heart failure and the associated remodeling. The signaling pathway consisted of myostatin, the protein kinase B, known as AKT, and the FOXO transcription factors FOXO1 and FOXO3. AKT in its active, phosphorylated state (*p*-AKT) is able to translocate to the nucleus, where it phosphorylates and therefore inactivates FOXO transcriptions factors [4]. FOXO1 and FOXO3 in their non-phosphorylated, active form induce the genes encoding for MAFbx and MuRF1 [4,5,6,7]. Especially FOXO3 is able to inhibit or reverse myocardial hypertrophy [8]. Thus, a disturbance of the myostatin/AKT/FOXO pathway could lead to an altered expression of MAFbx and MuRF1. Léger and colleagues stated that the inhibition of E3 ligases via the myostatin/AKT/FOXO pathway may protect against a total protein breakdown [9]. Further, this pathway has been shown to be responsible for a decrease in MAFbx and MuRF1 expression and the prevention of muscle atrophy [6,10,11]. An altered expression of the E3 ligases MAFbx and MuRF1 could also affect cardiac remodeling. 

Because the myostatin/AKT/FOXO pathway probably provides a new therapeutic target for cardiomyopathies, and the present findings gained from in vitro transgene expression studies [12], animal models [10,11,13,14,15,16,17] or from studies investigating skeletal muscle atrophy [5,6,9,10], we investigated the expression of molecules of the myostatin/AKT/FOXO pathway and the expression of MAFbx and MuRF1 in cardiomyopathy in humans. The aim of the study was to clarify if an upstream pathway that regulates the expression of MAFbx and MuRF1 is changed in dilated cardiomyopathy of ischemic (IDCM) and non-ischemic origin (NIDCM). According to the different etiologies of IDCM and NIDCM, we investigated both types of cardiomyopathies. 

## 2. Materials and Methods

### 2.1. Sample Collection and Preparation

The study was conducted in accordance with the Declaration of Helsinki and was approved by the Institutional Review Board of the Medical Faculty at the University of Leipzig (approval no.: 240/16-ek, Date: 17 October 2017). All patients provided written informed consent. Myocardial tissue was obtained during implantation of left ventricular assist device (LVAD) or during Morrow resection. Tissue from LVAD patients was obtained from the apex of 26 patients with IDCM and 23 patients with NIDCM. Septal tissue from 17 patients without diagnosed heart failure undergoing Morrow resection functioned as a control. Samples were dissected, snap-frozen in liquid nitrogen, and stored at −80 °C or fixed in 4% formaldehyde/phosphate-buffered saline for further analyses. 

### 2.2. RNA Isolation and cDNA Synthesis

RNA was isolated from snap-frozen myocardial tissue using the TRIzol Reagent (Thermo Fisher Scientific Inc., Waltham, MA, USA). After incubation of tissue in 1 mL TRIzol Reagent, addition of 200 µL chloroform and centrifugation at 4 °C and 12,000 rcf for 15 min, the aqueous phase was transferred into 500 µL isopropanol. Following centrifugation, the supernatant was discarded, and the RNA-containing pellet was washed in 1 mL 75% ethanol. The RNA-containing pellet was air-dried and then resuspended in 50 µL diethyldicarbonat (DEPC)-treated water (Thermo Fisher Scientific Inc.). The reverse transcription was performed according to the manufacturer instructions using the Applied Biosystems^TM^ High Capacity cDNA Reverse Transcription Kit (Thermo Fisher Scientific Inc.). In brief, 2 µL 10× RT Buffer, 0.8 µL 25× dNTP, 2 µL 10× RT random primers, 1 µL MultiScribe^TM^ Reverse Transcriptase, 1 µL RNase inhibitor and 3.2 µL DEPC-treated water were mixed with 10 µL RNA. After centrifugation, the reverse transcription mix was incubated in the peqSTAR Thermocycler (VWR International, Darmstadt, Germany) for 10 min at 25 °C and 120 min at 37 °C. The concentration and purity of the nucleic acids was determined using the Infinite 200 PRO microplate reader and the i-control^TM^ 1.12 software (both Tecan Trading AG, Männedorf, Switzerland).

### 2.3. Quantitative Real-Time Polymerase Chain Reaction

Quantitative real-time polymerase chain reaction (qRT-PCR) was utilized to quantify mRNA levels of MAFbx, MuRF1, FOXO1, FOXO3, AKT and myostatin with the QuantiNova SYBR^®^ Green PCR Kit (Qiagen, Hilden, Germany). The reaction mix consisted of 6.2 µL DEPC-treated water, 1.4 µL of forward and reverse primers, 10 µL SYBR Green PCR Master Mix and 1 µL cDNA. The PCR routine included an initial activation of the DNA polymerase for 2 min at 95 °C followed by 45 cycles of denaturation of the DNA strand for 10 s at 95 °C and combined primer annealing and extension for 20 s at 60 °C. All qRT-PCR reactions were performed in duplicate using the LightCycler^®^ 480 II and the LightCycler^®^ 480 SW 1.5 (both Roche Molecular Systems Inc., Basel, Switzerland). The primer sequences read as follows: MAFbx forward 5′-ACATGTGGGTGTATCGGATGG-3′, reverse 5′-GCACAAAGGCAGGTCAGTGA-3′; MuRF1 forward 5′-GCCCTGAGAGCCATTGACTT-3′, reverse 5′-GGCCTCTCATTCATCCAGCT-3′; FOXO1 forward 5′-ACCAAAGCTTCCCACACAGT-3′, reverse 5′-CTGCTTCTCTCAGTTCCTGCT-3′; FOXO3 forward 5′-AGTGTTGTGGAGAGCTGAGAC-3′, reverse 5′-TCACTACCAGATTCTCGGCTG-3′; AKT forward 5′-CCATGAAGACCTTTTGCGGC-3′, reverse 5′-CGACCGCACATCATCTCGTA-3′; myostatin forward 5′-TCCCAGGACCAGGAGAAGAT-3′, reverse 5′-TGCTCATCACAGTCAAGACCA-3′ and RPL4 forward 5′-CCAGGGTGCTTTTGGAAAC-3′, reverse 5′-AGATGGCGTATCGTTTTTGG-3′. Data were normalized to the housekeeping gene ribosomal protein L4 (RPL4), and ΔCt values were used to quantify relative gene expression levels. Results are expressed as mean of 2^–∆Ct^ ± standard deviation.

### 2.4. Protein Extraction and Quantification

Snap-frozen tissue (50 mg) was dispersed in 500 µL lysis buffer containing 90 mM HEPES, 126 mM KCl, 36 mM NaCl, 1 mM MgCl_2_, 50 mM EGTA, 8 mM ATP, 10 mM creatinphosphate and 1× Protease/Phosphatase Inhibitor Cocktail (Thermo Fisher Scientific Inc.) using a T25 digital ULTRA-TURRAX (IKA^®^—Werke GmbH & Co., KG, Staufen, Germany). Afterwards, the samples were sonicated for two cycles of 5 min with a 5 min break in an ultrasound water bath. The supernatants were collected after centrifugation at 14,000 rcf for 10 min at 4 °C. The protein concentrations were determined using the Pierce^TM^ Microplate BCA Protein Assay Kit (Thermo Fisher Scientific Inc.), the Infinite 200 PRO microplate reader and the i-control^TM^ 1.12 software.

### 2.5. Enzyme-Linked Immunosorbent Assays

Protein levels of MAFbx, MuRF1, FOXO1, FOXO3 and *p*-AKT (Phospho-S473) were quantified using enzyme-linked immunosorbent assay (ELISA) kits (Antibodies-online GmbH, Aachen, Germany and BIOZOL, Eching, Germany). The quantification of the myostatin protein levels was performed using the Quantikine ELISA (R&D Systems, Minneapolis, NE, USA). All ELISAs were performed according to the manufacturer instructions. The absorbances were measured at 450 nm and 570 nm (reference wavelength) using the Infinite 200 PRO microplate reader and the i-control^TM^ 1.12 software.

### 2.6. Immunohistochemistry

The immunohistochemical analysis of MAFbx and MuRF1 expression was performed using 3 µm sections of formaldehyde-fixed tissue. Sections were deparaffinized in xylole; 100%, 96%, 70% ethanol; and aqua dest. before equilibrating in tris-buffered saline (TBS; 20 mM Tris, 150 mM NaCl, pH 7.6). Permeabilization of the sections was induced by cooking with 0.01 M Na-citrate for 30 min. After cooling down, endogenous peroxidases were blocked with 60% methanol/40% TBS/0.1% hydrogen peroxide for 50 min. Sections were washed three times with TBS, and blocked with 2% bovine serum albumin (BSA)/TBS for 60 min before adding the primary antibodies detecting MAFbx (Proteintech, Munich, Germany) and MuRF1 (Abcam, Cambridge, UK). MAFbx and MuRF1 antigen recognition was visualized with a biotinylated secondary antibody (Sigma-Aldrich, St. Louis, MO, USA), streptavidin–horseradish peroxidase (Pierce @ Thermo Fisher Scientific, Waltham, MA, USA) and AEC staining solution (Sigma-Aldrich). Nuclei were counterstained with hemalaun for 5 min. The microscopic evaluation was performed with the Axioplan 2 microscope and the AxioVision Release 4.8.2 Sp3 software (both Carl Zeiss AG, Oberkochen, Germany).

### 2.7. Statistical Analysis

For statistical analysis IBM SPSS STATISTICS 28 (IBM Corporation, Armonk, New York, NY, USA) was consulted. Data are expressed as mean ± standard deviation. *p*-Values ≤ 0.05 were acknowledged as significant. The Levene test was used to analyze homoscedasticity of the groups. In case of equal variances, differences between the groups were examined by ANOVA for global analysis and by Scheffe test for post hoc analysis. In case of unequal variances, the Welch test carried out differences between the groups for global testing, and Dunnett-T3 was performed for post hoc analysis. To examine comparability of nominal variables, Pearson’s chi-squared test was used. In case of expected frequencies < 5, Fisher’s exact test was applied.

## 3. Results

### 3.1. Patient Characteristics

Demographic data and clinical characteristics of the patients are shown in Table 1. The study groups were comparable regarding sex, age at surgical intervention, body mass index (BMI), nicotine abuse and arterial hypertension.

### 3.2. Analysis of the mRNA Expression

**Figure 1:**
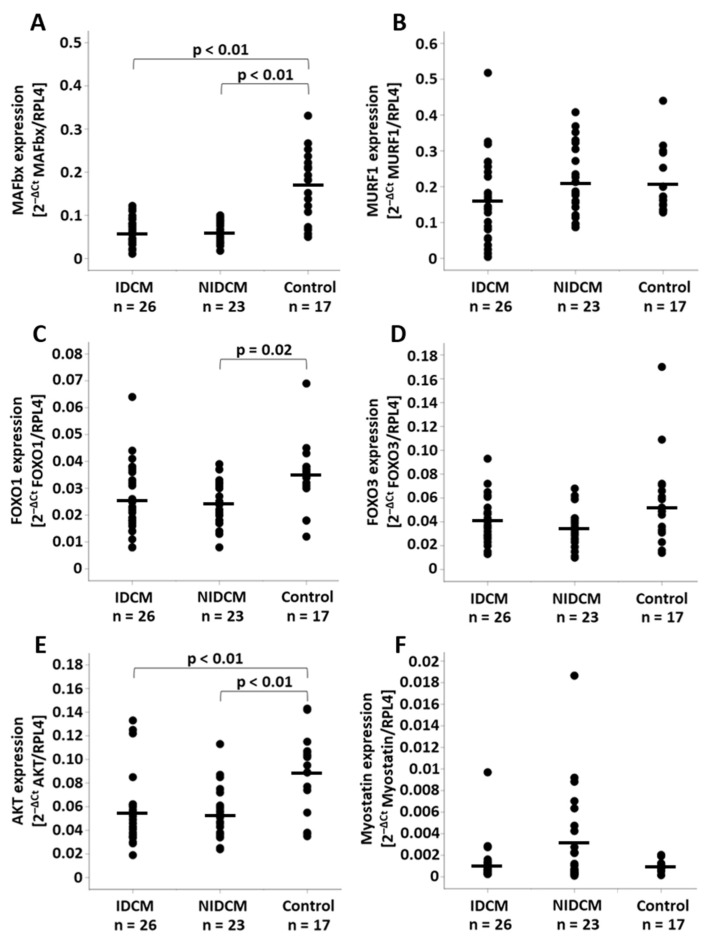
Relative mRNA expression of (**A**) MAFbx, (**B**) MuRF1, (**C**) FOXO1, (**D**) FOXO3, (**E**) AKT and (**F**) myostatin normalized to RPL4 expression in IDCM, NIDCM and control patients. One-way ANOVA was performed to compare the means of the study groups. Significant *p*-values of post hoc comparisons are shown. FOXO1, forkhead box protein O1; FOXO3, forkhead box protein O3; IDCM, dilated cardiomyopathy of ischemic origin; MAFbx, muscle atrophy F-box gene; MuRF1, muscle ring-finger protein-1; NIDCM, dilated cardiomyopathy of non-ischemic origin; RPL4, ribosomal protein L4.

MAFbx expression rates were reduced in IDCM (0.063 ± 0.031, *p* < 0.01) and NIDCM (0.064 ± 0.024, *p* < 0.01) compared to control patients (0.169 ± 0.080) (Figure 1A). No significant differences of MuRF1 expression rates could be identified among the groups (IDCM: 0.165 ± 0.117, NIDCM: 0.209 ± 0.098, control: 0.206 ± 0.087, *p* = 0.26) (Figure 1B). The analysis of the transcription factor FOXO1 revealed decreased mRNA levels in NIDCM (0.024 ± 0.008, *p* = 0.02) in comparison to control (0.035 ± 0.013). This difference was not observed in IDCM tissue (0.026 ± 0.013, *p* = 0.06) (Figure 1C). FOXO3, another transcription factor of MAFbx and MuRF1, was similar between IDCM (0.041 ± 0.019), NIDCM (0.034 ± 0.015) and control (0.056 ± 0.038, *p* = 0.06) patients (Figure 1D). Compared to the control group (0.089 ± 0.033), AKT gene expression rates were decreased in IDCM (0.056 ± 0.029, *p* < 0.01) and NIDCM (0.054 ± 0.021, *p* < 0.01) (Figure 1E). The expression of myostatin, an inhibitor of AKT in cardiomyocytes, did not differ between the groups (IDCM: 0.0012 ± 0.0019, NIDCM: 0.0033 ± 0.0044, control: 0.0009 ± 0.0005, *p* = 0.06) (Figure 1F).

### 3.3. Analysis of the Protein Expression

**Figure 2:**
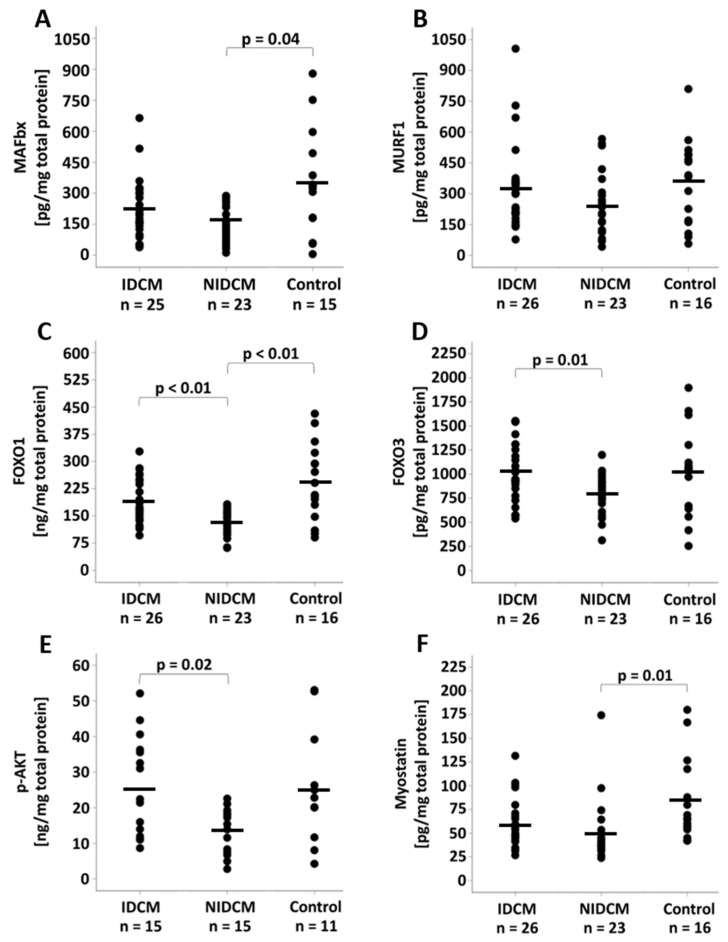
Protein expression of (**A**) MAFbx, (**B**) MuRF1, (**C**) FOXO1, (**D**) FOXO3, (**E**) AKT and (**F**) myostatin in IDCM, NIDCM and control patients. One-way ANOVA was performed to compare the means of the study groups. Significant *p*-values of post hoc comparisons are shown. FOXO1, forkhead box protein O1; FOXO3, forkhead box protein O3; IDCM, dilated cardiomyopathy of ischemic origin; MAFbx, muscle atrophy F-box gene; MuRF1, muscle ring-finger protein-1; NIDCM, dilated cardiomyopathy of non-ischemic origin.

While the protein expression of the E3 ligase MAFbx was reduced in NIDCM (166.4 ± 90.9 pg/mg, *p* = 0.04) when compared to control patients (349.1 ± 250.1 pg/mg), the MuRF1 protein expression did not differ between the groups (IDCM: 328.6 ± 205.2 pg/mg, NIDCM: 239.7 ± 154.9 pg/mg, control: 355.4 ± 207.4 pg/mg, *p* = 0.13) (Figure 2A,B). The immunohistochemical evaluation revealed that the proportion of MAFbx-expressing cells was comparable between the groups (IDCM: 37.8% ± 6.9%, NIDCM: 39.5% ± 5.4%, control: 40.8% ± 5.9%, *p* = 0.33). Comparisons of the groups for FOXO1 and FOXO3 protein expression showed lower protein levels in NIDCM (FOXO1: 132.5 ± 34.0 ng/mg, FOXO3: 797.3 ± 202.6 pg/mg) than in IDCM patients (FOXO1: 189.1 ± 61.5 ng/mg, FOXO3: 1026.4 ± 300.2 pg/mg, *p* < 0.01) (Figure 2C,D). Furthermore, the FOXO1 protein expression was decreased in NIDCM patients when compared to control patients (240.9 ± 105.2 ng/mg, *p* < 0.01). Protein expression levels of *p*-AKT differed between IDCM (25.9 ± 13.9 ng/mg) and NIDCM patients (13.8± 6.3 ng/mg, *p* = 0.02), and myostatin showed reduced protein levels in NIDCM patients (48.9 ± 31.8 pg/mg, *p* = 0.01) compared with control patients (82.2 ± 43.2 pg/mg) (Figure 2E,F).

## 4. Discussion

The inhibition or the reversal of cardiac remodeling in IDCM or NIDCM would be an important milestone to decelerate or treat cardiomyopathies. The development of new treatment strategies for cardiomyopathies requires the understanding of the involved pathophysiological processes. Based on the findings of Léger et al. examining the skeletal muscle [9], the present study investigated the myostatin/AKT/FOXO pathway and the expression of MAFbx and MuRF1 in IDCM and NIDCM to evaluate whether the upstream pathway, which regulates the expression of both E3 ligases, is changed in chronic heart failure and can therefore serve as a potential drug target to treat cardiomyopathies. The investigations showed that remarkable alterations have been observed in NIDCM but not in IDCM patients compared to controls. MAFbx and FOXO1 mRNA and protein expression as well as AKT mRNA and myostatin protein expression were decreased in NIDCM patients. Evaluating whether the myostatin/AKT/FOXO pathway is responsible for the downregulation of MAFbx suggests a cause-and-effect link between FOXO1 and MAFbx, because a diminished availability of FOXO1, a verified transcription factor of MAFbx [7], ultimately leads to a reduced abundance of this E3 ligase. The role of AKT and myostatin as upstream regulators of FOXO transcription factors is validated [8,14] but remains uncertain to cause alterations in FOXO1 and MAFbx in this case since we could not observe an upregulation of *p*-AKT protein levels that could explain the decreased active, non-phosphorylated FOXO1 levels. Consequently, it must be considered that the regulation of MAFbx in NIDCM could be AKT-independent. Moreover, no significant alterations in FOXO3 levels have been detected. Our results are in accordance with studies reporting the involvement of FOXO1 but not FOXO3 in the development of cardiac remodeling [8,18]. FOXO1 is known to induce cardiac cell death and to promote heart failure following cardiomyopathy [8]. A decreased expression of FOXO1, as it has been found in the NIDCM cohort of this study, could be interpreted as a rescue mechanism to avoid an enormous loss of cardiac cell mass and to slow down heart failure progression. This hypothesis has been reported in rodent animal models showing that FOXO1 deficiency prevented cardiac dysfunction [15,19]. Thus, it would be of interest to investigate if FOXO1 inhibition decelerates heart failure progression.

Our data showed that MAFbx, but not MuRF1 mRNA and protein expression, is downregulated in NIDCM. The E3 ligases MAFbx and MuRF1 regulate cardiac atrophy through proteasomal degradation of calcineurin and protein kinase C [20]. Both molecules, calcineurin and protein kinase C, mediate hypertrophic signaling, and their reduction prevents from the activation of hypertrophic pathways [16,17]. In contrast to hypertrophic cardiac remodeling, atrophic cardiac remodeling requires MAFbx which has been proven in MAFbx-/- mice that exhibited increased calcineurin expression and protein synthesis rates [17]. Additionally, Léger et al. detected decreased mRNA and protein levels of MAFbx in the skeletal muscle in a cohort of chronic spinal-cord-injured patients and assumed a protective mechanism that inhibits the persistent loss of muscle proteins [9]. Our results support these findings. Further, it can be suggested that a decreased MAFbx expression in the cardiac muscle may prevent the accumulation of polyubiquitinated proteins or their excessive degradation because both processes seem to be a part of the NIDCM pathology [21,22,23]. The reduction of cardiac-specific E3 ligases can affect downstream processes in the UPS, e.g., through producing less ubiquitin-marked proteins [23,24]. The findings of Spänig et al. substantiate these assumptions by showing a lower number of ubiquitin-positive cells in NIDCM compared to controls using immunohistochemistry [25].

In contrast to our results in NIDCM patients, analyses of the myostatin/AKT/FOXO pathway in IDCM tissue did not reveal significant alterations in comparison to controls: Apart from decreases of AKT and MAFbx mRNA expression, no significant differences were detected. These results are consistent with the report by Spänig et al., who did not show significant differences for MAFbx and MuRF1 in IDCM patients compared to controls [25]. Previous studies reported contrary results in less advanced stages of IDCM or after myocardial infarction, displaying opposing alterations in this pathway. Galasso et al. detected an increased protein expression of MAFbx and FOXO3 using Western blot in patients receiving coronary artery bypass surgery [26]. In contrast, Conraads et al. found a reduced MuRF1 and MAFbx mRNA and protein expression in biopsies from patients with myocardial infarction [27]. Our results revealed that the protein expression of members of the myostatin/AKT/FOXO pathway and the E3 ligases MAFbx and MuRF1 are unaffected in IDCM patients.

Comparing the groups for patient characteristics revealed a significantly higher percentage of patients suffering from type 2 diabetes mellitus in the IDCM group. Diabetes mellitus is known to influence FOXO expression and FOXO-related proteins. Perry et al. reported a reduced activity of AKT and an upregulated protein expression of FOXO1 in the skeletal muscle of diabetic patients, which could enhance the transcription of the E3 ligases MAFbx and MuRF1 [28]. These examinations could further influence our results in the IDCM group by antagonizing the protective downregulation of MAFbx and MuRF1.

Our study is limited by the following points: First, we used ELISAs and immunohistochemistry to quantify the protein expression of the targets of interest. A verification of the results by Western blot is preferred. Second, the study did not include the analysis of additional transcription factors of MAFbx and MuRF1. Therefore, the causal connection between the alterations of the myostatin/AKT/FOXO pathway and changes in MAFbx and MuRF1 expression cannot be asserted with absolute certainty. Third, data about genetic causes of NIDCM were not considered but could possibly influence our findings. Fourth, our control group did not consist of healthy patients. The advantage of including control patients that do not suffer from structural or ischemic heart disease is the higher availability. In this study, cardiac tissue of 17 control patients was included, while studies with organ-listed controls included lower numbers of patients, e.g., *n* = 4 in the study of Castillero et al. [29] or *n* = 5 in the study of George et al. [30].

If the reduction of MAFbx and FOXO1 mRNA and protein expression in NIDCM but not in IDCM patients is a rescue mechanism needs to be clarified in further studies. It would be interesting to know if the myostatin/AKT/FOXO pathway is responsible for the myocardial recovery that has been reported in non-ischemic forms of cardiomyopathy [31,32].

## 5. Conclusions

In summary, our results demonstrate that the myostatin/AKT/FOXO pathway is altered in NIDCM but not in IDCM patients. FOXOs, especially FOXO1, seem to be an important drug target for regulating the expression of MAFbx in NIDCM patients. Future studies will show whether FOXO1 inhibition is suitable to treat NIDCM.

## Figures and Tables

**Table 1:** Demographic data and clinical characteristics of the study groups.

	IDCM (*n* = 26)	NIDCM (*n* = 23)	Control (*n* = 17)	*p* Value
Male sex	17 (65.4%)	12 (52.2%)	8 (47.1%)	0.45
Age at surgical intervention [years]	65.5 ± 6.4	66.7 ± 8.3	69.2 ± 7.9	0.28
BMI [kg/m^2^]	28.2 ± 5.4	27.5 ± 6.0	30.2 ± 4.8	0.29
LVEF [%]	22.0 ± 6.7	21.6 ± 5.3	63.1 ± 9.7	<0.01
NT-proBNP [ng/L]	9042 ± 8996	10,488 ± 16,700	-	0.36
NYHA classification				<0.01
Class I	1 (3.8%)	0 (0%)	4 (23.5%)	
Class II	3 (11.5%)	1 (4.3%)	8 (47.1%)	
Class III	7 (26.9%)	17 (73.9%)	6 (29.4%)	
Class IV	15 (57.7%)	5 (21.7%)	0 (0%)	
Nicotine abuse				0.07
Smoker	8 (30.8%)	2 (8.7%)	0 (0.0%)	
Non-smoker	12 (46.2%)	13 (56.5%)	13 (76.5%)	
Ex-smoker	5 (19.2%)	8 (34.8%)	4 (23.5%)	
Unknown	1 (3.8%)	0 (0%)	0 (0%)	
Arterial hypertension	24 (92.3%)	16 (69.6%)	13 (76.5%)	0.12
Coronary heart disease	26 (100%)	3 (13.0%)	5 (29.4%)	<0.01
Type 2 diabetes	15 (57.7%)	7 (30.4%)	3 (17.6%)	0.02
Myocardial infarction	24 (92.3%)	0 (0%)	0 (0%)	<0.01
Renal function eGFR [mL/min/1.73 m^2^] Creatinine [µmol/L]	43.9 ± 19.7 159.4 ± 80.1	45.1 ± 20.8 158.2 ± 112.5	78.1 ± 17.9 79.3 ± 27.2	<0.01 <0.01

BMI, body mass index; eGFR, estimated glomerular filtration rate; IDCM, dilated cardiomyopathy of ischemic origin; LVEF, left ventricular ejection fraction; NIDCM, dilated cardiomyopathy of non-ischemic origin; NT-proBNP, N-terminal pro B-type natriuretic peptide; NYHA, New York Heart Association.

## Data Availability

The data presented in this study are available on request under consideration of ethical regulations from the corresponding author.
